# Supplementary material for: The interactome of intact mitochondria by cross-linking mass spectrometry provides evidence for coexisting respiratory supercomplexes
Source: Mol Cell Proteomics. 2017 Dec 8;17(2):216–32. doi: 10.1074/mcp.RA117.000470 (PMC5795388; doi:10.1074/mcp.RA117.000470)
Supplement: Supplemental Data [file supp_RA117.000470_133922_0_supp_23978_fzffwf.zip › spectra_annotation/mito_spectra_annotation/15-1-9-1-5-1.pdf]

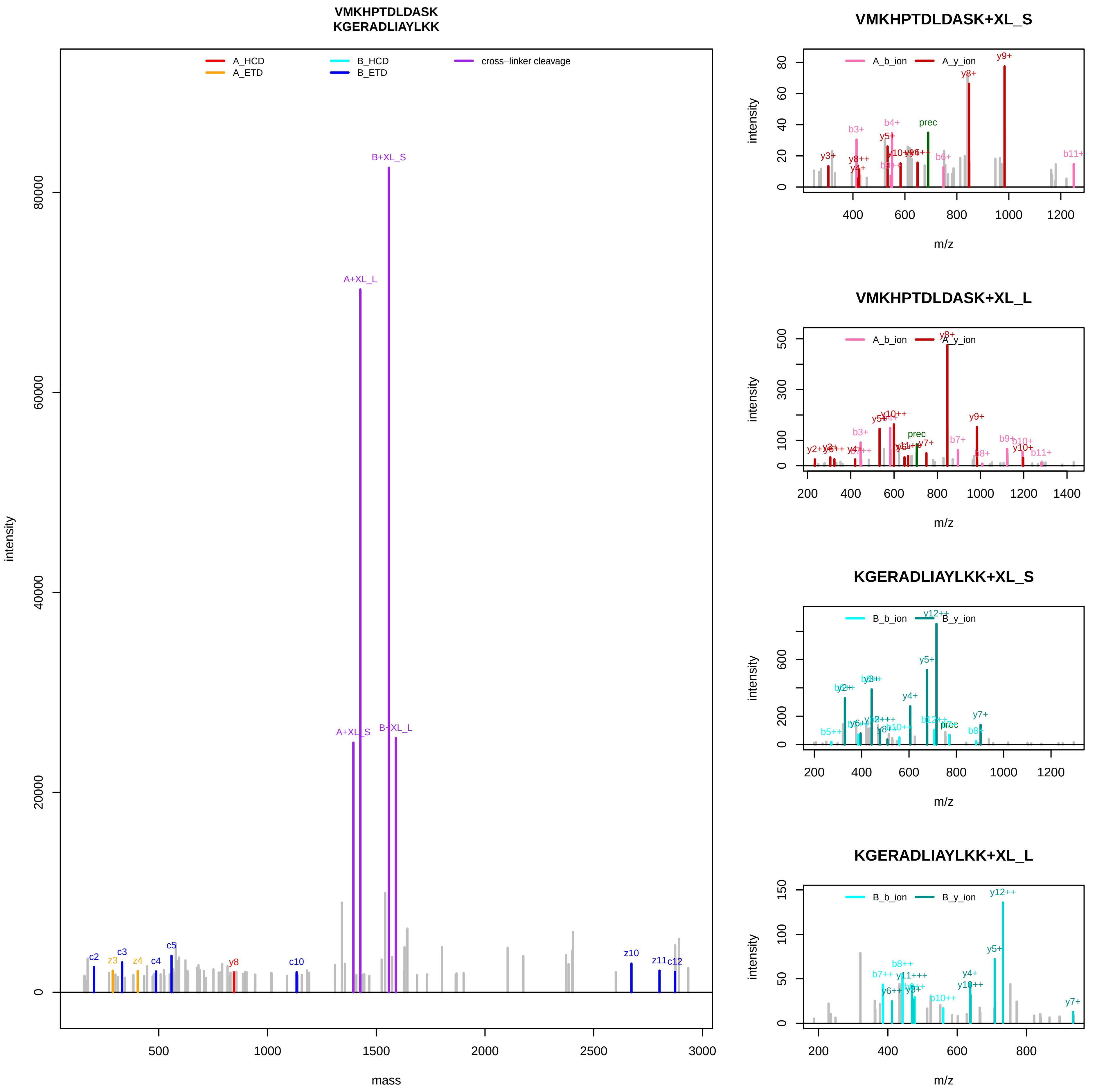
VMKHPTDLDASK
KGERADLIAYLKK
A_HCD
A_ETD
B_HCD
B_ETD
cross−linker cleavage
B+XL_S
A+XL_L
A+XL_S
B+XL_L
c2
z3
c3
z4
c4
c5
y8
c10
z10
z11
c12
intensity
mass
0
20000
40000
60000
80000
500
1000
1500
2000
2500
3000
VMKHPTDLDASK+XL_S
A_b_ion
A_y_ion
y9+
y8+
b3+
b4+
y5+
prec
y3+
y8++
y4+
b6+
b11+
intensity
m/z
VMKHPTDLDASK+XL_L
y8+
y9+
b3+
prec
y7+
b7+
b9+
b10+
y10+
b11+
y10++
y4+
KGERADLIAYLKK+XL_S
B_b_ion
B_y_ion
y12++
y5+
y3+
y2+
y4+
y7+
b5++
b8+
prec
KGERADLIAYLKK+XL_L
y12++
y5+
y4+
y10++
y7+
b8++
b7++
y11+++
y6++
b10++
200
400
600
800
1000
1200
1400
